# Toosendanin suppresses African swine fever virus replication through upregulating interferon regulatory factor 1 in porcine alveolar macrophage cultures

**DOI:** 10.3389/fmicb.2022.970501

**Published:** 2022-08-30

**Authors:** Yuanjia Liu, Xinheng Zhang, Zexin Liu, Li Huang, Weixin Jia, Xinlei Lian, Changjiang Weng, Guihong Zhang, Wenbao Qi, Jianxin Chen

**Affiliations:** ^1^Guangdong Provincial Key Laboratory of Veterinary Pharmaceutics Development and Safety Evaluation, College of Veterinary Medicine, South China Agricultural University, Guangzhou, China; ^2^College of Animal Science, South China Agricultural University, Guangzhou, China; ^3^State Key Laboratory of Veterinary Biotechnology, Harbin Veterinary Research Institute, Chinese Academy of Agricultural Sciences, Harbin, China; ^4^African Swine Fever Regional Laboratory of China (Guangzhou), College of Veterinary Medicine, South China Agricultural University, Guangzhou, China

**Keywords:** African swine fever virus, toosendanin, antiviral activity, porcine alveolar macrophages, interferon regulatory factor 1

## Abstract

African swine fever virus (ASFV) is a highly infectious and lethal swine pathogen that causes severe socio-economic consequences in affected countries. Unfortunately, effective vaccine for combating ASF is unavailable so far, and the prevention and control strategies for ASFV are still very limited. Toosendanin (TSN), a triterpenoid saponin extracted from the medicinal herb *Melia toosendan* Sieb. Et Zucc, has been demonstrated to possess analgesic, anti-inflammatory, anti-botulism and anti-microbial activities, and was used clinically as an anthelmintic, while the antiviral effect of TSN on ASFV has not been reported. In this study, we revealed that TSN exhibited a potent inhibitory effect on ASFV GZ201801-38 strain in porcine alveolar macrophages (PAMs; EC_50_ = 0.085 μM, SI = 365) in a dose-dependent manner. TSN showed robust antiviral activity in different doses of ASFV infection and reduced the transcription and translation levels of ASFV p30 protein, viral genomic DNA quantity as well as viral titer at 24 and 48 h post-infection. In addition, TSN did not affect virion attachment and release but intervened in its internalization in PAMs. Further investigations disclosed that TSN played its antiviral role by upregulating the host IFN-stimulated gene (ISG) IRF1 rather than by directly inactivating the virus particles. Overall, our results suggest that TSN is an effective antiviral agent against ASFV replication *in vitro* and may have the potential for clinical use.

## Introduction

African swine fever (ASF) is a highly contagious viral disease caused by African swine fever virus (ASFV) infection, with morbidity and mortality rates close to 100%. It is a devastating disease in domestic and wild pigs, causing heavy losses to the pig and related industries in affected countries because of a wide variety of transmission routes and a lack of effective vaccines and anti-ASFV drugs (Liu et al., 2021). ASF was first reported in Kenya in 1921 and sustained epidemics circulated in the continent for 36 years. In 1957, ASF first crossed Africa and broke out in Portugal and then underwent several important intercontinental transmissions and spread to Russia, Ukraine, Belarus, Lithuania, Estonia, Poland, Latvia, Romania, the Czech Republic, and Hungary (Penrith and Vosloo, 2009; Netherton et al., 2019). In August 2018, the first case of ASF in China was confirmed in Shenyang City, Liaoning Province (Ge et al., 2018; Zhou et al., 2018a). Then, ASFV spread across the country, reducing the pig population (Wang et al., 2018; Liu et al., 2020b) and causing a heavy loss to the pig industry because of the limited prevention and control strategies.

ASFV is a large enveloped DNA arbovirus, and the only member of the *Asfarviridae* family, *Asfivirus* genus (Costard et al., 2009). The viral genome of ASFV consists of a central conserved region and two-variable ends (Alkhamis et al., 2018). The length of ASFV genomic ranges between 170 and 193 kilobases (kb) depending on different isolates and codes for between 150 and 167 proteins, including those required for virus replication (Dixon et al., 2013). The main structural component of the viral capsid is protein p72 (Munoz-Moreno et al., 2015). P30, p54, and p72 are rich structural proteins in ASFV virion, and their expression level can represent the amount of virus to some extent (Fan et al., 2020). Macrophages are the primary target cells for ASFV replication, which (the process) play a key role in activating innate and adaptive immune responses to antagonize ASFV replication (Galindo and Alonso, 2017; Dixon et al., 2019).

Vaccines and drugs are two essential tools for controlling infectious disease. However, for ASFV, its large genome and complex immune escape mechanism pose a great challenge to developing efficient ASFV vaccines, and no safe and effective commercial vaccine against ASFV has been released so far (Zhuo et al., 2021). Early detection, elimination of the infectious source and enhanced biosecurity measures are major measures to control its spread (Liu et al., 2021). In this context, efforts to seek new strategies for ASFV prevention and control are critical, including the development of safe and efficient anti-ASFV drugs. In fact, attempts for developing anti-ASFV drugs have been made for decades, and some compounds were reported to possess anti-ASFV activity in cell cultures (Arabyan et al., 2018, 2019; Hakobyan et al., 2019). However, the effective *in vitro* anti-ASFV concentrations of these compounds are too high, and the *in vivo* anti-ASFV efficacy of these compounds has not been validated. Therefore, the search for new effective antiviral agents to control ASFV is urgently needed (Huang et al., 2021).

Toosendanin (TSN), a triterpenoid saponin extracted from the medicinal herb *Melia toosendan* Sieb. et Zucc by Chinese scientists in 1950, has been used as agricultural insecticide for decades and was ever used as an ascarifuge in China (Shi and Li, 2007; Ma et al., 2013). TSN has been demonstrated to possess analgesic, anti-inflammatory, anti-botulism and anti-microbial activities (He et al., 2010; Jin et al., 2017). Besides, TSN has been reported that possess anti-proliferative and apoptosis-inducing effects on various human cancer cells *in vitro*. For examples, it was reported that TSN inhibits tumor cells growth by inducing apoptosis in colorectal cancer through suppression of the AKT/GSK-3β/β-catenin pathway (Wang et al., 2015). TSN can induce caspase-dependent apoptosis through activating the p38 MAPK pathway in human gastric cancer cells (Zhou et al., 2018b). In addition, TSN can inhibit adipogenesis by activating Wnt/β-catenin signaling (Chen et al., 2018). However, little research has been documented on the antiviral activity of TSN. In 2011, Watanabe et al. demonstrated that either TSN alone or a combination of TSN and IFNα could inhibit hepatitis C virus (HCV) infection in a human hepatoma cell line (Huh7; Watanabe et al., 2011). In 2019, Jin et al. reported that TSN inhibits influenza A virus infection at an early stage by altering PA protein nuclear localization (Jin et al., 2019). In 2021, Li et al. revealed a broad anti-viral effect of TSN to bunyaviruses and the emerging SARS-CoV-2 (Li et al., 2021). However, antiviral effect of TSN on ASFV has not been reported.

In our anti-ASFV drug screening test, TSN exhibited the most potent antiviral activity among 1,260 tested compounds. In the present study, the antiviral effects of TSN against ASFV replication in PAMs and involved mechanisms were comprehensively investigated.

## Materials and methods

### Cells and viruses

Porcine alveolar macrophages (PAMs) were obtained from 4-week-old specific-pathogen-free pigs used for ASFV genotype II strain replication in this study (Carrillo et al., 1994; Borca et al., 2020). Briefly, the lungs were washed three times with cooled phosphate-buffered saline (PBS) containing penicillin (300 IU/ml) and streptomycin (300 μg/ml). Cells were centrifuged at 800 *g* for 10 min, resuspended in RPMI 1640 (Gibco, Waltham, MA, United States) supplemented with 10% fetal bovine serum (FBS; Gibco) and 100 IU/ml penicillin and 100 μg/ml streptomycin at 1 × 10^6^ cells/ml in a 6-well plate, and then incubated at 37°C for 2 h. The suspending cells (mainly lymphocytes and red blood cells) were removed, and adherent cells were PAMs. PK15 cells were purchased from American Type Culture Collection (ATCC). PK-15 cells were maintained in Dulbecco’s Modified Eagle’s Medium (DMEM; Thermo Fisher Scientific, Waltham, MA, United States) supplemented with 10% fetal bovine serum (FBS; Gibco) in a 37°C incubator with 5% CO_2_.

ASFV genotype II GZ201801 isolate was used in this study, and it is preserved in the African Swine Fever Regional Laboratory of China at South China Agricultural University. ASFV GZ201801-38 strain, obtained from the GZ201801 by continuous culture in PAMs for 38 generations, which was well adapted to PAMs and could infect PK15 cells maintaining low levels of replication. PK-15 cells were cultured in 96-well plates and infected with ten-fold diluted ASFV. Viruses were detected by the Indirect Immunofluorescence assay (IFA) method and then the viral titers were calculated using the Reed Muench method (Reed and Muench, 1938). In this study, the experiments involving ASFV were carried out in a biosafety level-3 laboratory at South China Agricultural University (Guangzhou, China).

### Cytotoxicity assay

The cytotoxicity of TSN was evaluated using the MTT assay. Briefly, 2 × 10^5^ PAMs (per well) were seeded in 96-well plates and seven concentrations of the drug intervention group (27, 9, 3, 1, 0.33, 0.11, and 0.04 μM TSN), solvent group and blank control group (mock) were set up with five replicate wells in each group. It was then incubated at 37°C for 3 h. Then serially diluted TSN or 2% DMSO or empty medium were added to the cells in each group, and the cells were incubated for 48 h. Then the culture medium was removed and replaced with 100 μl of 3-(4,5-dimethylthiozol-2-yl)-3,5-dipheryl tetrazolium bromide (MTT; Sigma-Aldrich) solution (0.5 mg/ml in PBS) and incubated at 37°C for 4 h. The wells were added with 150 μl DMSO to dissolve the formazan crystals for 10 min at 37°C after removal of the supernatant. Cell viability was measured as the absorbance at 490 nm with a microplate reader (Thermo fisher scientific, MA, United States) and expressed as a percentage of the control well. The mean optical density (OD) values from five wells per treatment were used as the cell viability index. The 50% cytotoxic concentration (CC_50_) was analyzed by GraphPad Prism 5.0 (GraphPad Software, San Diego, CA, United States).

### Indirect immunofluorescence assay

The PAMs in different treatment groups were fixed with 4% paraformaldehyde for 30 min, then washed three times by PBS, permeabilized with 0.3% Triton-X100 solution per well for 15 min at room temperature, blocked with 1% bovine serum albumin (BSA) for 1 h at room temperature and then incubated with a mouse monoclonal antibody against the p30 protein of ASFV (1:3,000 dilution) at 4°C overnight. After being washed three times with PBS, the cells were incubated for 1 h at room temperature with a goat anti-mouse secondary antibody conjugated with AlexaFluor^®^ 488 (Green; 1:1,000 dilution, Cell signaling Technology, MA, United States). Nuclei were counterstained using 50 μl of 4,6-diamidino-2-phenlindole (DAPI, 300 nM; sigma-Aldeich; blue). Immunofluorescence was captured using the Leica DMI 4000B fluorescence microscope (Leica, Wetzlar, Germany). The fluorescence densities (blue fluorescence and green fluorescence) of each well were digitalized by ImageJ software. The negative control group was set to 100%, and the other groups were compared with the negative control group. The EC_50_ value was determined by quantifying the protection rate of compound treatment and calculated by nonlinear regression function using GraphPad Prism 7.0 software (Su et al., 2021).

### Reverse transcription quantitative PCR and quantitative PCR

Total DNA was extracted from the PAMs in different treatment groups using the FastPure^®^ Cell/Tissue DNA Isolation Mini Kit (Vazyme Biotech, China, Nanjing) and total RNA was extracted from the cells using the RNA fast 200 kits (Fastagen, China, Shanghai) according to the manufactures’ instructions. The extracted DNA was further used to determine the DNA level of ASFV replication, and the extracted RNA was further used to determine the RNA level of ASFV replication. For the extracted RNA, it was further reverse transcribed into cDNA using a reverse transcription kit (Genstar, Beijing, China) according to the manufacturer’s instructions. The obtained cDNAs or extracted DNA were used as templates for RT-qPCR and qPCR, respectively, on a CFX96 real-time polymerase chain reaction system (Bio-Rad, Hercules, CA, United States) using 2 × RealStar Green Power Mixture (containing SYBR Green I Dye; Genstar, Beijing, China). The primers used for determining the DNA level of ASFV replication by using p72 gene (Nunez-Hernandez et al., 2018) were listed in Table 1. The primers (Arabyan et al., 2018) used for determining the mRNA level of ASFV replication by using the p72 and p30 genes were listed in Table 2. The primers used for determining the mRNA level of 9 ISGs and IFNs were listed in Table 3. In qPCR results, Log_10_ (viral genomic copies) was shown on Y-axis, which was calculated by the standard curve (*y* = −2.812*x* + 13.45, *R*^2^ = 0.999) and this formula: (6.02 × 10^23^) × (ng/μl × 10^−9^)/(DNA length×660) = copies/μl. Relative mRNA expression was calculated by the 2^∆∆CT^ method. GAPDH was used as the endogenous control (Liu et al., 2020a).

**Table 1:** The primers used for determining the DNA level of ASFV replication by using p72 gene by qPCR.

Name	Sequence (5′–3′)
p72-F	GCAAAGACTGAACCCACTAATTT
p72-R	TGTCATCATATTTGGCAGGTTT

**Table 2:** The primers used for determining the mRNA level of ASFV replication by using p30 and p72 genes by qRT-PCR.

Name	Sequence (5′–3′)
RT-p30-F	TGCACATCCTCCTTTGAAACAT
RT-p30-R	TCTTTTGTGCAAGCATATACAGCTT
RT-p72-F	ACGGCGCCCTCTAAAGGT
RT-p72-R	CATGGTCAGCTTCAAACGTTTC
GAPDH-F	CCTTCCGTGTCCCTACTGCCAAC
GAPDH-R	GACGCCTGCTTCACCACCTTCT

**Table 3:** The primers used for determining the mRNA level of 9 ISGs and IFNs by qRT-PCR.

Name	Sequence (5′–3′)
IFNα-F	AGAAGCATCTGCAAGGTTCC
IFNα-R	AGATGGCATTGCAGCTGAGTA
IFNβ-F	CCAGCAGATCTTCGGCATTC
IFNβ-R	AGGTCATCCATCTGCCCATC
IFNγ-F	GATCCAGCGCAAAGCCATCA
IFNγ-R	TCTGGCCTTGGAACATAGTCTG
TRIM26-F	GAGGCCTGCGAGAATTCCAA
TRIM26-R	GTAGGTCACGCACTTCCAGT
IRF1-F	GCAACAGATGAGGACGAG
IRF1-R	GCTTTCAACTTCTGGCTC
SAT1-F	TCGGAGAGCACCCCTTCTAC
SAT1-R	TGGCAAAACCAACAATGCTGT
ISG20-F	TACCATCTACGACACCGCCC
ISG20-R	AAAGTTCCATTGTTGCCCTGG
MX1-F	TACGACATCGAATACCAGATCAA
MX1-R	ATGGTCCTGTCTCCTTCGG
PKR-F	ATTGCGAGAAGGTAGAGCGT
PKR-R	TTCCATTTGGATGAAAAGGCACC
GBP1-F	GAAGGGTGACAACCAGAACGAC
GBP1-R	AGGTTCCGACTTTGCCCTGATT
DCP1A-F	ATGGAGTCGCTGAGTCGAG
DCP1A-R	CTGGTGATGTAGGGGTCGTG
SHFL-F	ATGAAGCAGGACCGTGACAT
SHFL-R	ACATGCGTAGGTTGGCTTCT

### Endpoint dilution method

PK15 cells were seeded in 96-well plates and incubated at 37°C. Test group and blank control group were set up with five replicate wells in each group. When the cell monolayer was grown to 80%–90% confluency, cells in the test group were infected with ten-fold serially diluted samples for 2 h, then the supernatant was discarded and cells were washed by PBS, fresh medium was added to the cells in the test and blank control groups, followed by additional incubation at 37°C for 48 h. Viruses were detected by the Indirect Immunofluorescence assay (IFA) method and then TCID_50_ was calculated using the Reed Muench method (Reed and Muench, 1938).

### Western blot

The PAMs in different treatment groups were lysed in RIPA lysis buffer containing 1 mM phenylmethylsulfonylfluoride (Beyotime, Shanghai, China) at 4°C. The supernatant was harvested by centrifugation at 10,000 *g* for 20 min at 4°C, and the total protein for each sample was measured using the BCA protein assay kit. The supernatant was mixed with 5 × SDS PAGE-loading buffer and boiled for 10 min. The protein samples were separated in 12% SDS-PAGE and transferred onto a PVDF membrane. The membrane was blocked for 1 h at room temperature in 5% milk prepared in TBS/Tween 20 (0.1%) and incubated with monoclonal or polyclonal antibody of each protein overnight at 4°C. After being washed with TBST, the membrane was then incubated with appropriate goat anti-mouse IgG (H + L)-HRP for 1 h at 37°C. Finally, the blots were visualized using the enhanced chemiluminescence (ECL) reagent (Cwbiotech Company, Beijing, China). Mouse anti-GAPDH monoclonal antibody (1:1,000 dilution), rabbit anti-α-Tubulin monoclonal antibody (1:1,000 dilution) were purchased from Beyotime (Shanghai, China). Rabbit anti-IRF1 polyclonal antibody (1:1,000 dilution) were purchased from Proteintech Group, Inc. (Rosemont, United States). Rabbit anti-FLAG monoclonal antibody (1:1,000 dilution) was purchased from Invitrogen (Thermo Fisher Scientific, Inc., PA). ASFV p30 polyclonal antibody (1:3,000 dilution), Goat anti-rabbit IgG-HRP (1:3000 dilution) and goat anti-mouse IgG-HRP (1:3,000 dilution) were purchased from Beyotime (Shanghai, China). The GAPDH and a-Tubulin were used as the endogenous control.

### Antiviral activity assay

The antiviral activity assay for TSN was performed to evaluate its inhibiting capacity on ASFV *in vitro*. PAMs that seeded in 24-well, or 48-well plates were infected with ASFV (0.014 MOI) for 2 h at 37°C. The supernatant was removed, and fresh maintenance medium (RPMI 1640 with 2% FBS) containing different concentrations of TSN was added. Cells and supernatants were then collected at the indicated time points post-infection and subjected to three freeze–thaw cycles at −80°C and 4°C, respectively, to ensure the maximal release of cellular virions. Viral titer was determined by the endpoint dilution method and expressed as log_10_ TCID_50_/ml. Viral DNA, RNA and protein levels were determined using qPCR, RT-qPCR and Western Blot, respectively. Viral p72 gene was used to determine ASFV DNA level, while viral p30 gene was used to determine ASFV mRNA level and protein level.

### ASFV binding assay

The PAMs in the treatment group and negative control group were pre-chilled at 4°C for 15 min. ASFV was diluted by medium, which was pre-chilled at 4°C, and then TSN was diluted by 3 times with ASFV solution to obtain a mixture of virus and TSN at 1 μM, or 0.33 μM, or 0.11 μM. The mixture containing 0.014 MOI ASFV was used to inoculate PAMs in the treatment and negative groups. After incubating at 4°C for 2 h, the supernatant was discarded and PAMs was washed by PBS and incubated in fresh medium. At 24 hpi, the samples were subjected to qPCR, RT-qPCR and endpoint dilution method analysis.

### ASFV internalization assay

The PAMs grown in 48-well plates were infected with 0.014 MOI ASFV and incubated at 4°C for 2 h. After 2 h, ASFV were removed and PAMs were incubated in fresh medium containing different concentrations of TSN (1, 0.33, and 0.11 μM) or without TSN. After 3 h incubation at 37°C, the supernatant was discarded and PAMs were washed by PBS and incubated in fresh medium for additional 24 h, then the samples were subjected to qPCR, RT-qPCR and endpoint dilution method analysis.

### ASFV release assay

The PAMs seeded in 48-well plates were infected with ASFV (0.014 MOI) for 2 h at 37°C. After 2 h, the cells were washed by PBS and then replaced with fresh medium. After cultured for another 19 h, the cells were washed with PBS and incubated in fresh medium containing different concentrations of TSN (1, 0.33, and 0.11 μM) or without TSN. After culturing for another 6 h, the supernatant was collected for qPCR and endpoint dilution method analysis.

### TSN pretreatment

PAMs were incubated in medium containing 1 μM of TSN at 37°C for 3, or 6, or 9, or 12 h, then washed by PBS. The cells were infected with ASFV (0.014 MOI) for 2 h and collected at 24 hpi for qPCR and RT-qPCR analysis. For investigating the preventive effects of different doses of TSN, PAMs were incubated in medium containing different concentrations of TSN (1, 0.33, and 0.11 μM) for 6 h. After three washes by PBS, the cells were infected with ASFV (0.014 MOI) for 2 h and collected at 24 hpi for qPCR, RT-qPCR and endpoint dilution method analysis.

### Co-treatment of TSN and ASFV

The PAMs were seeded in 48-well plates and incubated at 37°C for 3 h. ASFV was diluted with medium, and TSN was diluted into 1, 0.33, and 0.11 μM using the virus solution, respectively. The mixture of TSN and ASFV (0.014 MOI) was then added to the treatment group, while the negative control group was added with the TSN-free virus solution. The cells in different groups were incubated for 2 h at 37°C, and then washed by PBS, and then added fresh medium. At 24 hpi, the cells were collected for qPCR, RT-qPCR and endpoint dilution method analysis.

### Time-of-addition and time-of-elimination assay

The PAMs were seeded in 48-well plates and incubated at 37°C for 3 h. The cells were infected with 100 μl of ASFV (0.014 MOI) at 37°C for 2 h, then the supernatant was discarded and the cells were washed by PBS. For the time-of-addition assay, fresh medium containing 1 μM of TSN was added to the cells in the treatment group at 3, 6, 9, 12, and 15 hpi, respectively. For the time-of-elimination assay, fresh medium containing 1 μM of TSN was added to the cells in the treatment group, and medium without TSN was added to the cells in the negative control group. The supernatant was discarded at 3, 6, 9, 12, and 15 hpi, respectively, and then added fresh medium to the cells. For the two experiments, the samples were collected at 24 hpi for qPCR and RT-qPCR analysis.

### ASFV-TSN direct interaction

Two hundred microliter of diluted ASFV (2 × 10^6^ TCID_50_/ml) was incubated with different concentrations of TSN (1, 0.33, and 0.11 μM) at 37°C for 2 h. ASFV diluted with the medium containing 2% DMSO was used as the control group. Then PAMs were treated with the mixture at 4°C for 2 h. After 2 h, the cells were washed by PBS followed by additional incubation at 37°C for 22 h, then the samples were collected for qPCR, RT-qPCR and endpoint dilution method analysis.

### RNA sequencing and transcriptome data analysis

8 × 10^5^ PAMs (per well) were seeded in 24-well plates and C group (mock group), CD group (The group of PAMs incubated with TSN), CV group (The group of ASFV-infected PAMs) and CVD group (The group of ASFV-infected PAMs incubated with TSN) were set up with three replicate wells in each group. After incubated at 37°C for 3 h. The cells in the CV and CVD group were infected with ASFV (0.014 MOI) at 37°C for 2 h, then the supernatant was discarded, fresh medium containing 1 μM of TSN was added to the cells in the CD and CVD groups, and fresh medium without TSN was added to the cells in the C and CV groups. 24 h later, the samples were collected, total RNA was isolated using the TRIzol reagent (Invitrogen, Carlsbad, CA, United States) according to the manufacturer’s recommendations.

Illumina sequencing was performed at Novogene Bioinformatics Technology Co., Ltd., in Beijing, China. Briefly, Sequencing libraries were generated using the NEBNext1 Ultra™ RNA Library Prep Kit for Illumina1 (NEB, United States), following the manufacturer’s protocol. The library fragments were purified with AMPure XP system (Beckman Coulter, Beverly, United States) and library quality was assessed on the Agilent Bioanalyzer 2100 system. The clustering of the index-coded samples was performed on a cBot Cluster Generation System using TruSeq PE Cluster Kit v3-cBot-HS (Illumia), the library preparations were sequenced on an Illumina Novaseq platform and 150 bp paired-end reads were generated. Raw data (raw reads) of fastq format were firstly processed through in-house perl scripts. Clean data (clean reads) were obtained by removing reads containing adapter, reads1 containing N base and low-quality reads from raw data. At the same time, Q20, Q30, and GC content the clean data were calculated. All the downstream analyses were based on the clean data with high quality. Index of the reference genome was built using Hisat2 v2.0.5 and paired-end clean reads were aligned to the reference genome using Hisat2 v2.0.5. We selected Hisat2 as the mapping tool for that Hisat2 can generate a database of splice junctions. FeatureCounts v1.5.0-p3 was used to count the reads numbers mapped to each gene. And then FPKM of each gene was calculated based on the length of the gene and reads count mapped to this gene. Differential expression analysis of two groups (two biological replicates per condition) was performed using the DESeq2 R package (1.20.0). DESeq2 provide statistical routines for determining differential expression in digital gene expression data using a model based on the negative binomial distribution. The resulting *p*-values were adjusted using the Benjamini and Hochberg’s approach for controlling the false discovery rate. Genes with an adjusted *p*-value <0.05 found by DESeq2 were assigned as differentially expressed.

### Knockdown of the endogenous IRF1 through RNA interference (RNAi)

Three pairs of siRNAs and a scrambled siRNA (siRNA-Scra) were designed by the GenePharma Company (Suzhou, China) and were used to silence the expression of IRF1 in PAMs (Table 4). PAMs were infected with ASFV (0.014 MOI) at 37°C for 2 h，and then transfected with the indicated siRNA using lipofectamine RNAiMAX (Invitrogen, Life Technologies, Carlsbad, CA) in serum-free Opti-MEM at 37°C for 4 h according to the manufacturer’s protocol (Liu et al., 2020a). Then the cells were treated with 1 μM of TSN at 37°C for 24 h, followed by Western blot and endpoint dilution method analysis.

**Table 4:** Three pairs of siRNAs designed to silence the endogenous IRF1.

Target	Designation	Sequence (5′–3′)	Orientation
PAMs-IRF1	NC	UUCUCCGAACGUGUCACGUTT	Sense
PAMs-IRF1		ACGUGACACGUUCGGAGAATT	Antisense
PAMs-IRF1	sscIRF1-301	GGGCUGAUCUGGAUUAAUATT	Sense
PAMs-IRF1		UAUUAAUCCAGAUCAGCCCTT	Antisense
PAMs-IRF1	sscIRF1-666	CCCUGAUACCUUCUCUGAUTT	Sense
PAMs-IRF1		AUCAGAGAAGGUAUCAGGGTT	Antisense
PAMs-IRF1	sscIRF1-980	AGACAAACGUGGAUGGGAATT	Sense
PAMs-IRF1		UUCCCAUCCACGUUUGUCUTT	Antisense

### Overexpression of IRF1

PK15 cells grown to 80%–90% confluence were transfected with pRK-5-FLAG-IRF1, using JetPRIME Polyplus-transfection reagent (PolyPlus Transfection, New York, NY) to confirm the expression of the plasmids *in vitro* (Liao et al., 2020). For each transfection, 2 μg DNA of plasmid was transfected into the PK15 cells in each well of a 6-well plate. Non-transfected cells were used as a negative control. Western blot was performed to confirm the expression of the recombinant plasmids.

### Statistical analysis

All experiments were performed in triplicate. All data are shown as mean ± SD. Statistical significance was determined by Student’s *t-*test when only two groups were compared or by one-way analysis of variance (ANOVA) when more than two groups were compared. ^*^p < 0.05, ^**^p < 0.01, and ^***^p < 0.001 were considered as statistically significant differences.

## Results

### TSN inhibits ASFV replication in PAMs with minimum cytotoxicity

**Figure 1:**
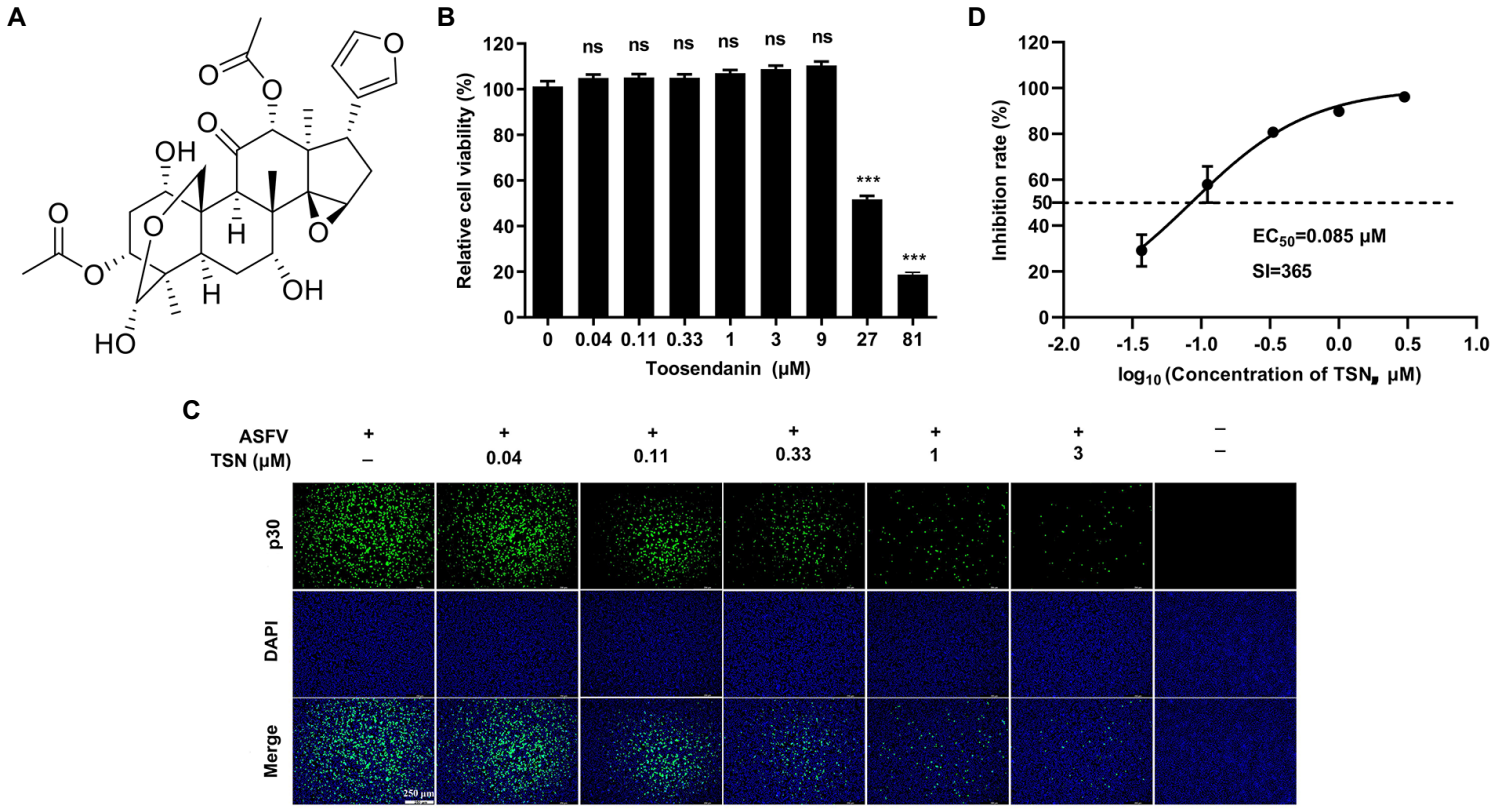
TSN inhibits ASFV infection in PAMs with no cytotoxicity. **(A)** Chemical structure of TSN. **(B)** Cytotoxicity of TSN on PAMs was analyzed by MTT assay as described in the Methods. **(C)** Antiviral activity of TSN against ASFV in PAMs was determined by IFA. Briefly, PAMs were seeded into 24-well plates，infected with ASFV (0.014 MOI) for 2 h and then incubated with maintenance medium containing indicated concentrations of TSN. ASFV was detected by immunofluorescence using a mouse anti-p30 antibody at 48 hpi. The nuclei were counter-stained with DAPI (blue). **(D)** EC_50_ of TSN in ASFV-infected PAMs was calculated as described in the Methods. ^***^ denotes *p* < 0.001; ns denotes no significant difference.

The chemical structure of TSN was shown in Figure 1A. We first tested the cytotoxicity of TSN in PAMs post-incubation for 48 h by MTT assay. The results showed that TSN did not impair PAMs viability at the concentrations ranging from 0.04 to 9 μM, the CC_50_ was 31 μM (Figure 1B). Next, we examined the antiviral effects of TSN at different concentrations against ASFV in PAMs at 48 hpi by IFA. As shown in Figure 1C, ASFV replication, reflected by the percentage of virus-infected PAMs, was significantly inhibited by TSN in a dose-dependent manner. The EC_50_ of TSN was calculated as 0.085 μM using the ImageJ software (Figure 1D), and thus the selective index (SI) of TSN was 365.

**Figure 2:**
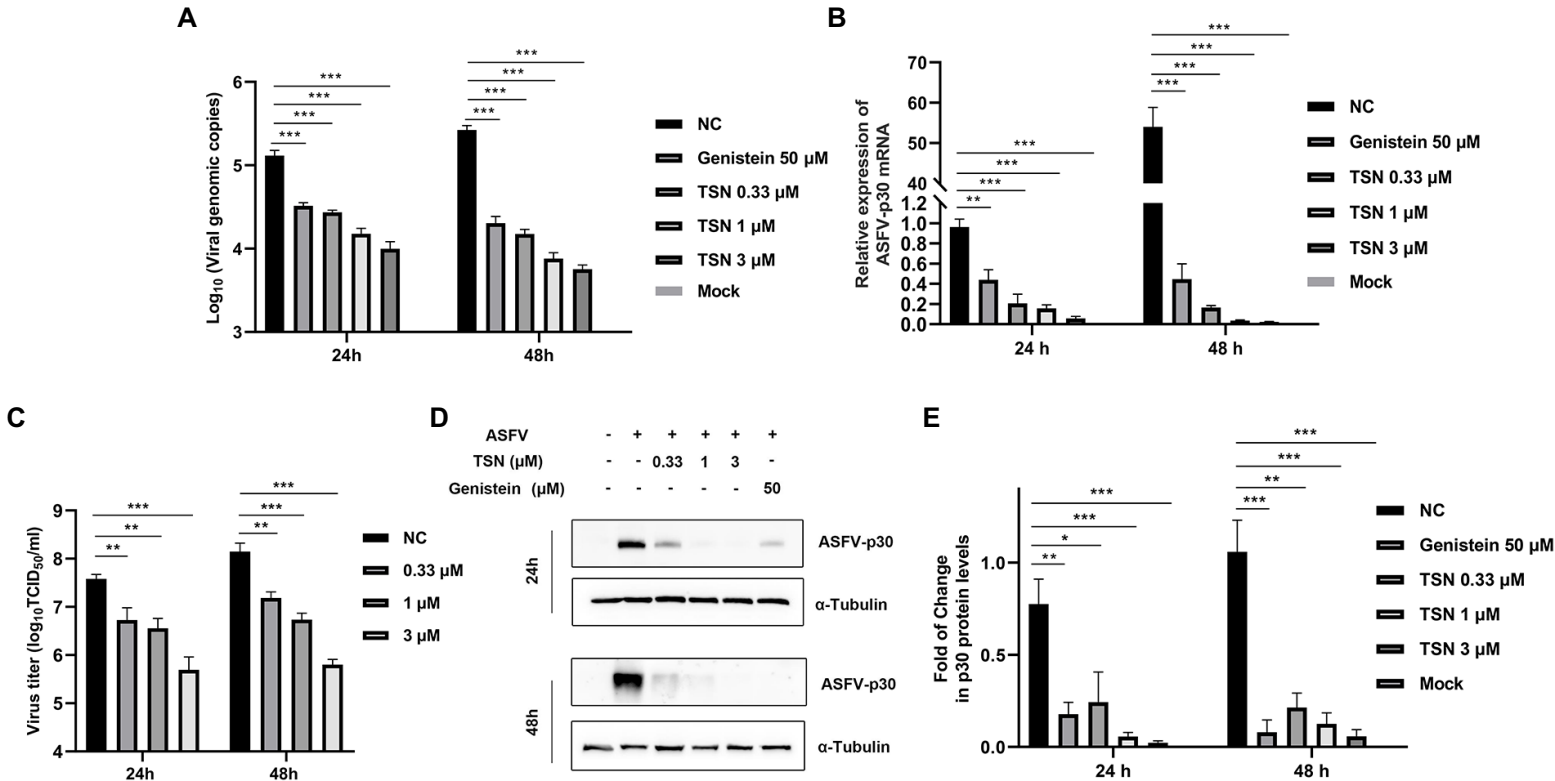
TSN exhibits continuous inhibition on ASFV replication in a dose-dependent manner in PAMs. PAMs seeded in 24-well or 48-well plates were infected with ASFV (0.014 MOI) at 37°C for 2 h and then washed with PBS and cultured in a fresh maintenance medium containing different concentrations of TSN. At 24 and 48 hpi, the samples were subjected to qPCR, RT-qPCR, Western Blot and viral titer analysis. **(A)** The DNA level of the ASFV p72 gene was analyzed using qPCR. **(B)** The mRNA level of the ASFV p30 gene was analyzed using RT-qPCR. **(C)** The ASFV titer was determined at 24 and 48 hpi using the endpoint dilution method as described in the Methods. **(D)** The expression of viral p30 protein in PAMs treated with TSN or Genistein was detected by Western Blot. Genistein (50 μM) was used as a positive control. **(E)** The densitometric analysis of p30 from **(D)** was performed using the ImageJ software and data were normalized by comparing to the virus control (NC) at 48 h (set as 1). ^***^ denotes *p* < 0.001; ^**^ denotes *p* < 0.01; ^*^ denotes *p* < 0.05. NC denotes the negative control (only ASFV infection without TSN treatment).

We further investigated the antiviral effect of TSN at different concentrations against ASFV. The DNA level of p72 gene of ASFV were dose-dependently reduced in different concentrations of TSN at 24 hpi and 48 hpi (Figure 2A). Similarly, TSN treatment also resulted in dose-dependently decrease of the mRNA level of the p30 gene (Figure 2B) and the virus titer of ASFV (Figure 2C). Consistently, results from Western blot analysis also confirmed that the viral p30 protein expression was dose-dependently reduced in different concentrations of TSN at 24 and 48 hpi, with unobservable level at 3 μM TSN (Figure 2D). A previous study showed that genistein is an effective agent for inhibiting ASFV (Arabyan et al., 2018). Therefore, genistein was used as the positive drug control for inhibiting ASFV in this study. Results in Figure 2D showed that the genistein could significantly suppress the expression of viral p30 protein at 24 and 48 hpi. The protein level of p30 at 24 and 48 hpi in different treatment groups were shown as fold of change in Figure 2E. These results together indicate that TSN significantly inhibits ASFV infection at sub-micromolar concentrations in PAMs in a dose-dependent manner.

### Antiviral effects of TSN against different ASFV challenge doses in PAMs

**Figure 3:**
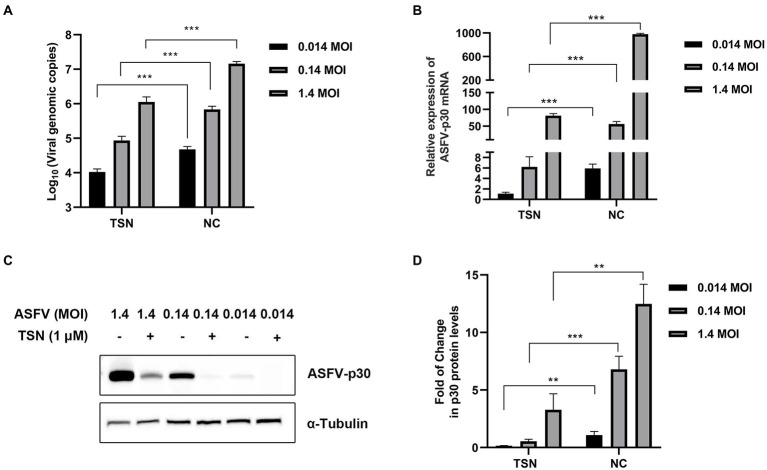
Antiviral effects of TSN against different challenge doses of ASFV in PAMs. PAMs seeded in 24-well or 48-well plates were challenged with different ASFV doses and treated with 1 μM TSN. The negative control was infected with ASFV without TSN treatment. The samples were subjected to qPCR, RT-qPCR, and Western Blot at 24 hpi. **(A)** The DNA level of the ASFV p72 gene was analyzed using qPCR. **(B)** The mRNA level of the ASFV p30 gene was analyzed using RT-qPCR. **(C)** The expression of viral p30 protein was detected by Western Blot. **(D)** The densitometric analysis of p30 from **(C)** was performed using the ImageJ software. ^***^ denotes *p* < 0.001; ^**^ denotes *p* < 0.01. NC denotes the negative control.

Next, we further evaluated the antiviral efficacy of TSN against different ASFV challenge doses in PAMs. As shown in Figure 3A, under the treatment of 1 μM TSN, the DNA level of ASFV p72 gene showed a dose-dependently decline at different challenge doses (1.4 MOI, 0.14 MOI, and 0.014 MOI) at 24 hpi in PAMs. Similarly, TSN treatment at 1 μM reduced the mRNA level of ASFV p30 in a dose-dependent manner at 24 hpi in PAMs (Figure 3B); As shown in Figures 3C,D, it also reduced ASFV p30-protein level in a dose-dependent manner at 24 hpi in PAMs. These results indicate that the inhibition of TSN on ASFV is independent of challenge dose.

### TSN suppresses ASFV infection at different treatment modes in PAMs

**Figure 4:**
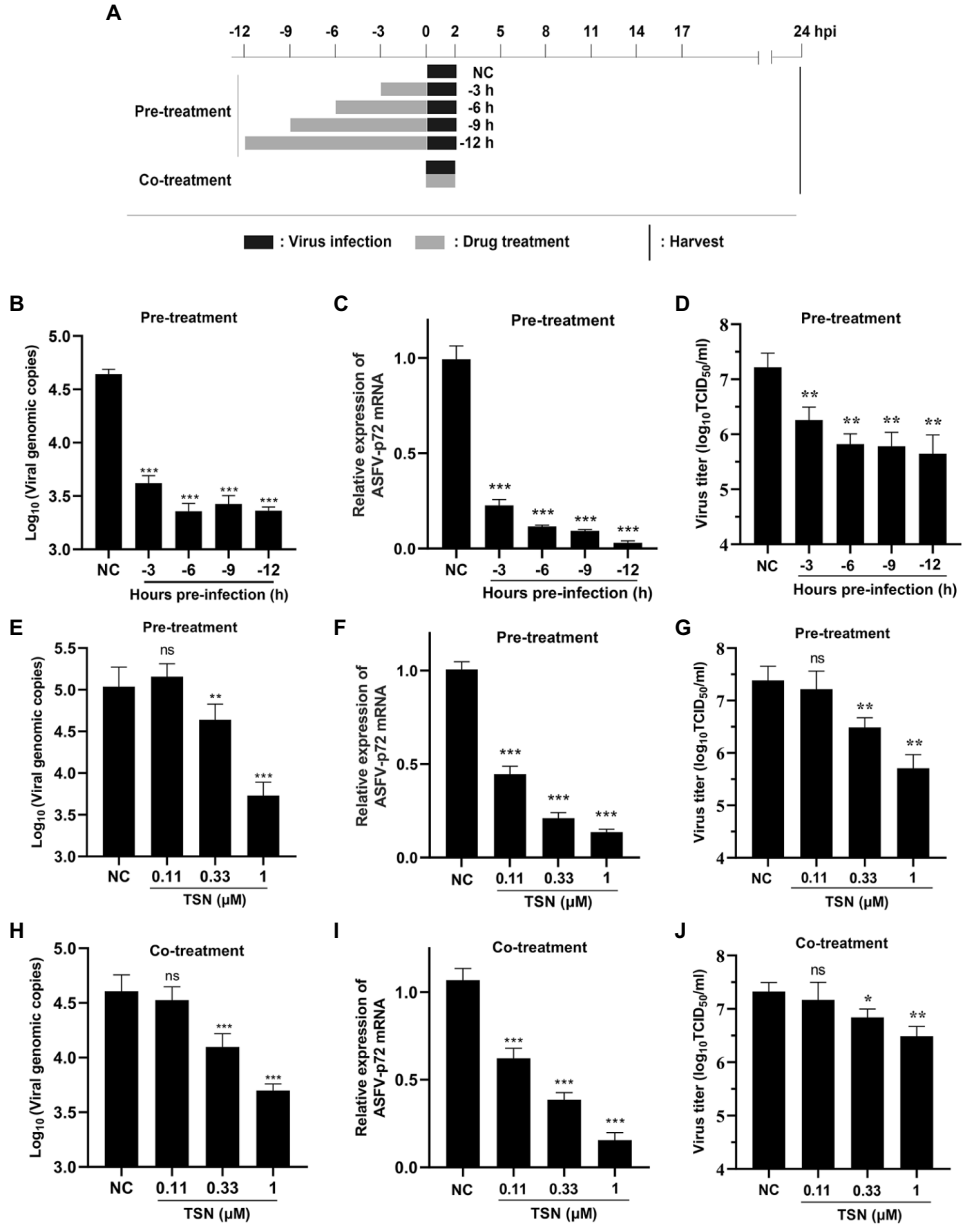
TSN suppresses ASFV replication in pre-and co-treatment modes in PAMs. **(A)** PAMs were seeded in 48-well plates for 3 h and then treated with TSN (1 μM) for 3–12 h prior to ASFV infection (pre-treatment), for 2 h during viral infection (co-treatment). For all two treatment modes, 0.014 MOI of ASFV GZ201801-38 was used to infect the PAMs for 2 h. After 24 hpi, the cells were subjected to qPCR or RT-qPCR. **(B–D)** For the pre-treatment experiment, the DNA level of ASFV p72 gene was determined by qPCR **(B)**, the mRNA level of ASFV p72 was determined by RT-qPCR **(C)** and the virus titer of ASFV was determined by endpoint dilution method **(D)**. **(E–G)** For the pre-treatment experiment, the DNA level of ASFV p72 gene was determined by qPCR **(E)**, the mRNA level of ASFV p72 was determined by RT-qPCR **(F)** and the virus titer of ASFV was determined by endpoint dilution method **(G)**. **(H–J)** For the co-treatment experiment, the DNA level of ASFV p72 gene was determined by qPCR **(H)**, the mRNA level of ASFV p72 was determined by RT-qPCR **(I)** and the virus titer of ASFV was determined by endpoint dilution method **(J)**. ^***^ denotes *p* < 0.001; ^**^ denotes *p* < 0.01; ns denotes no significant difference. NC denotes the negative control.

To explore the specific stage(s) of the ASFV replication cycle at which TSN exerted its antiviral effects, the PAMs were infected with ASFV for 2 h and TSN was added into the cell cultures before (Pre-treatment), during (Co-treatment) and post (Post-treatment) ASFV infection with different incubation time. The pattern diagram of pre-and co-treatment was shown in Figure 4A. The results of the pre-treatment experiment showed that TSN (1 μM) pre-treatment for 3, 6, 9, and 12 h significantly reduced the DNA level of p72 gene (Figure 4B) and the mRNA level of ASFV p72 (Figure 4C) and the virus titer of ASFV (Figure 4D). Besides, the results of pre-treatment of the different concentrations of TSN (1, 0.33, and 0.11 μM) showed that pre-treatment of 1 or 0.33 μM TSN for 6 h could significantly reduce the DNA level of p72 (Figure 4E) and the mRNA level of p72 gene (Figure 4F) and the virus titer of ASFV (Figure 4G). Interestingly, when the PAMs were treated with TSN (1, 0.33, and 0.11 μM) during ASFV infection (Co-treatment), the DNA level of the p72 gene (Figure 4H) and the virus titer of ASFV (Figure 4J) in 1 and 0.33 μM TSN treatment groups were all significantly decreased, it also resulted in a dose-dependently decrease in the mRNA level of p72 gene of ASFV (Figure 4I).

**Figure 5:**
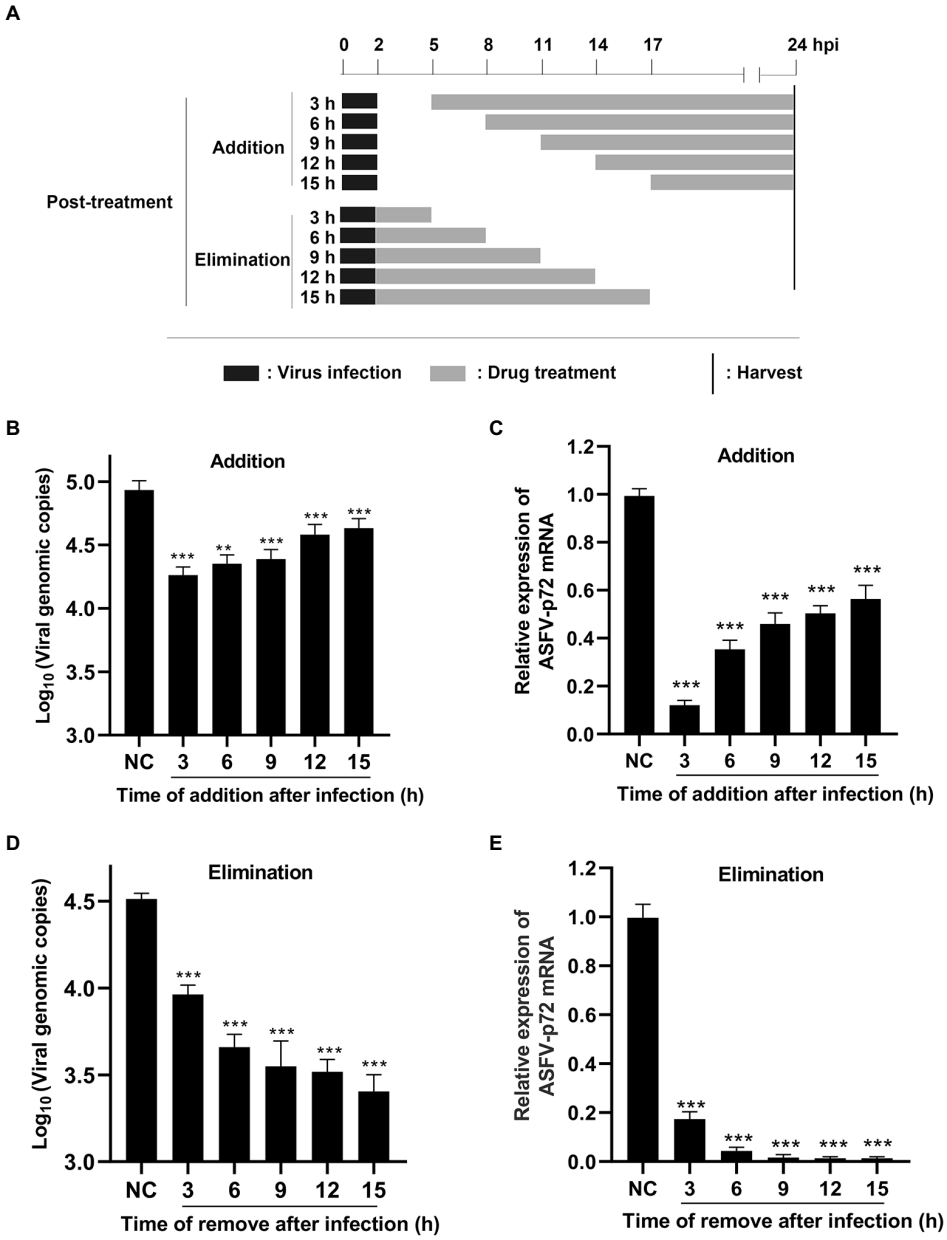
Post-treatment of TSN suppresses ASFV replication in PAMs. **(A)** For the post-treatment experiment, PAMs seeded in 48-well plates were challenged with ASFV (0.014 MOI) for 2 h. 1 μM TSN was added into the cell at 3, 6, 9, 12, and 15 hpi, respectively; For the elimination experiment, PAMs seeded in 48-well plates were challenged with ASFV (0.014 MOI) for 2 h, and then 1 μM TSN was added into the cells. TSN was then discarded at 3, 6, 9, 12 and 15 hpi, respectively, and replaced with fresh maintenance medium. All the samples of the two experiments were harvested at 24 hpi for qPCR and RT-qPCR. **(B, C)** The DNA level of ASFV p72 gene was determined by qPCR **(B)** and the mRNA level of ASFV p72 gene was determined by RT-qPCR **(C)**. **(D, E)** The DNA level of ASFV p72 gene was determined by qPCR **(D)** and the mRNA level of ASFV p72 was determined by RT-qPCR **(E)**. ^***^ denotes *p* < 0.001. NC denotes the negative control.

The pattern diagram of time-of-addition and time-of-elimination assay (post-treatment) was shown in Figure 5A. Results showed that TSN exerted the most significant inhibition on the DNA level of p72 when added at 3 hpi; with the delay of TSN added, the ability of TSN to inhibit ASFV decrease reflected by increasing trend of DNA level of p72 (Figure 5B). A similar trend was also observed in the mRNA level of p72 gene (Figure 5C). Meanwhile, the time-of-elimination assay was conducted to identify the inhibitory effects of TSN (1 μM) discarded at different time points post ASFV infection (3, 6, 9, 12, and 15 hpi). The results showed TSN treatment for only 3 h post-infection resulted in significant decrease in the DNA level of p72 gene (Figure 5D) as well as the mRNA level of p72 gene (Figure 5E), and the decreasing trend increased with the prolongation of TSN treatment. These results together suggests that in ASFV infected PAMs, the earlier of TSN added and the longer of TSN treatment, the stronger inhibitory effect TSN exhibits.

### TSN does not affect viral attachment and release but affects viral internalization in PAMs

**Figure 6:**
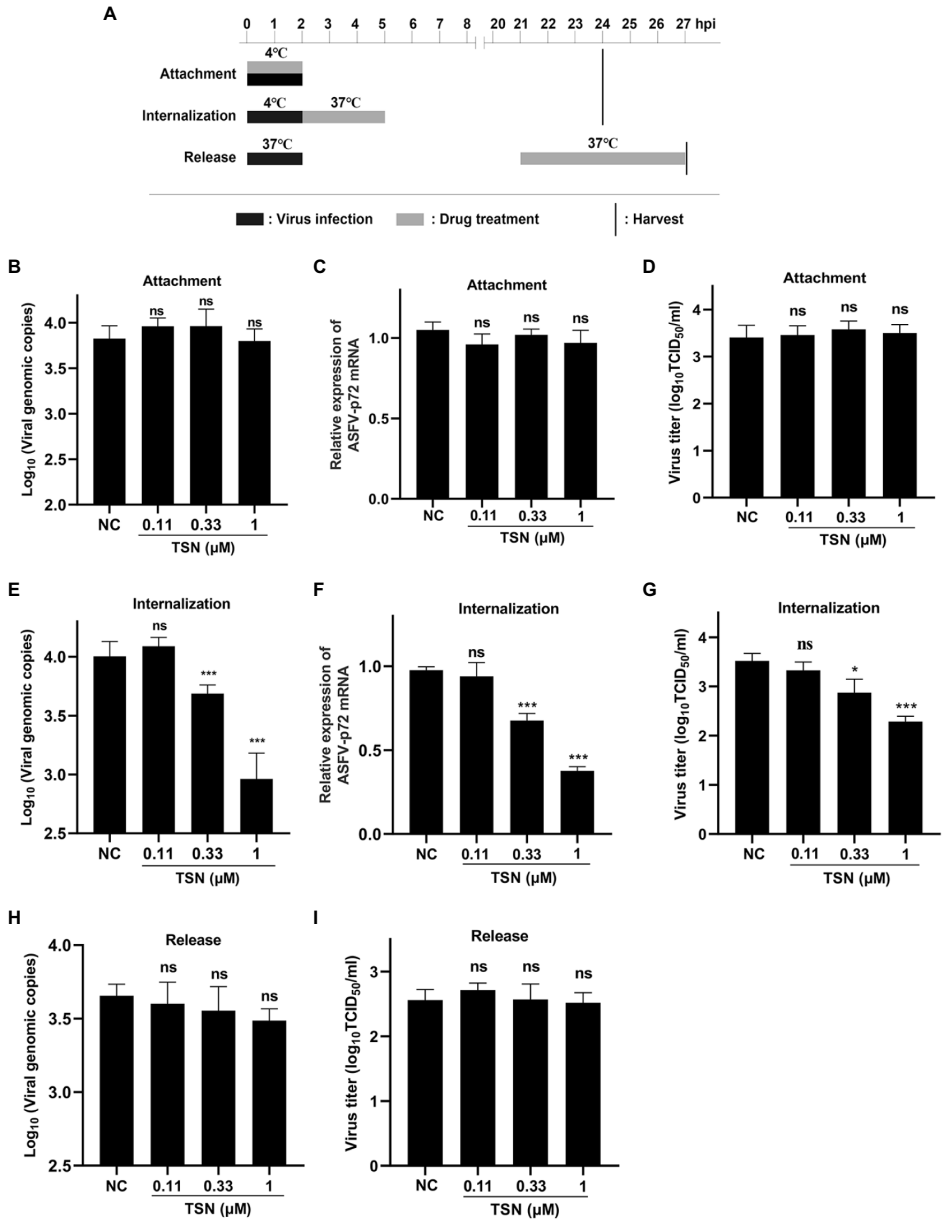
TSN does not affect viral attachment and release, while it affects viral internalization. **(A)** PAMs were seeded in 48-well plates. For attachment experiment, the mixture of TSN and ASFV was added to PAMs and incubated at 4°C for 2 h; For internalization experiment, PAMs were incubated by ASFV (0.014 MOI) at 4°C for 2 h, then 1 μM TSN was added to the wells and incubated at 37°C for 3 h; For releasement experiment, PAMs were incubated by ASFV (0.014 MOI) at 37°C for 2 h, then washed with PBS and replaced with fresh maintenance medium, 1 μM TSN was added to the wells at 21–27 hpi. All the samples were harvested at 24 or 27 hpi for qPCR and endpoint dilution method analysis. **(B)** Viral attachment was investigated by detecting the DNA level of ASFV p72 gene by qPCR. **(C)** Viral attachment was investigated by detecting the mRNA level of ASFV p72 by RT-qPCR. **(D)** Viral attachment was investigated by detecting the viral titer using endpoint dilution method as described in the Methods. **(E)** Viral internalization was investigated by detecting the DNA level of ASFV p72 gene by qPCR. **(F)** Viral internalization was investigated by detecting the mRNA level of ASFV p72 by RT-qPCR. **(G)** Viral internalization was investigated by detecting the viral titer using endpoint dilution method as described in the Methods. **(H)** Viral releasement was investigated by detecting the DNA level of ASFV p72 gene by qPCR. **(I)** Viral releasement was investigated by detecting the viral titer using endpoint dilution method as described in the Methods. ^*^ denotes *p* < 0.05; ^***^ denotes *p* < 0.001; ns denotes not significant. NC denotes negative control.

The pattern diagram of adsorption, internalization and release was shown in Figure 6A. To investigate the effect of TSN on viral binding to PAMs, viral internalizing and releasing from PAMs, the DNA level of the p72 gene and the mRNA level of the p72 gene in different TSN treatment modes were measured. As shown in Figures 6B,C, the results from the attachment experiment showed that the DNA level of ASFV p72 gene (Figure 6B) and the mRNA level of ASFV p72 (Figure 6C) and the virus titer of ASFV (Figure 6D) were not significantly affected by TSN treatment, indicating that TSN does not affect ASFV attachment to PAMs. Interestingly, the results from the internalization experiment showed that the TSN at 1 and 0.33 μM could significantly decrease the DNA level of p72 gene (Figure 6E), the mRNA level of p72 gene (Figure 6F) and the virus titer of ASFV (Figure 6G), indicating that TSN is able to intervene ASFV internalization in PAMs. In addition, the results from the ASFV release experiment showed that the DNA level of ASFV p72 was not affected by TSN treatment (Figure 6H), similarity, the virus titer of ASFV was not affected by TSN treatment (Figure 6I), indicating that TSN does not affect viral release from ASFV infected PAMs.

### TSN does not directly interact with ASFV in PAMs

**Figure 7:**
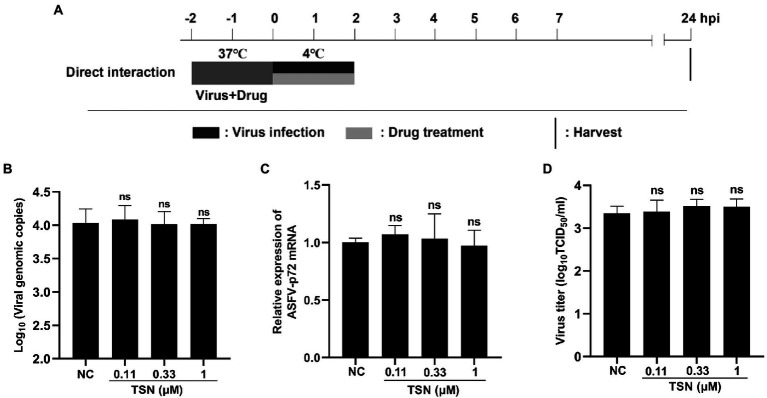
TSN has no direct inactivation on ASFV. **(A)** The mixture of ASFV (2 * 10^6^ TCID_50_/mL) and TSN (0.11, 0.33, and 1 μM) was incubated at 37°C for 2 h. Then the PAMs seeded in 48-well plates were challenged with the mixture and incubated at 37°C for 2 h. The supernatant was discarded and replaced with fresh maintenance medium. The samples were harvested at 24 hpi for qPCR, RT-qPCR and endpoint dilution method analysis. **(B)** The DNA level of the ASFV p72 gene was analyzed using qPCR. **(C)** The mRNA level of the ASFV p72 gene was analyzed using RT-qPCR. **(D)** Viral titer was investigated by endpoint dilution method as described in the Methods. ns denotes no significant difference. NC denotes the negative control.

As shown above, TSN exhibited strong inhibition on ASFV infection at different treatment modes and did not affect the attachment process of ASFV, which leads us to consider that TSN might play its inhibition *via* affecting cellular components or signaling pathway(s) rather than directly interacting with ASFV. To exclude the latter possibility, the direct interaction between TSN and ASFV was investigated. The pattern diagram of the direct action was shown in Figure 7A. As shown in Figures 7B–D, co-incubation of TSN (at 1, 0.33, and 0.11 μM) with ASFV did not affect the DNA level of the p72 gene (Figure 7B), the mRNA level of p72 gene (Figure 7C) and the virus titer of ASFV (Figure 7D), demonstrating that TSN does not directly interact with ASFV to affect viral replication.

### Effects of TSN on the mRNA expression level of host genes in PAMs by RNA sequencing

**Figure 8:**
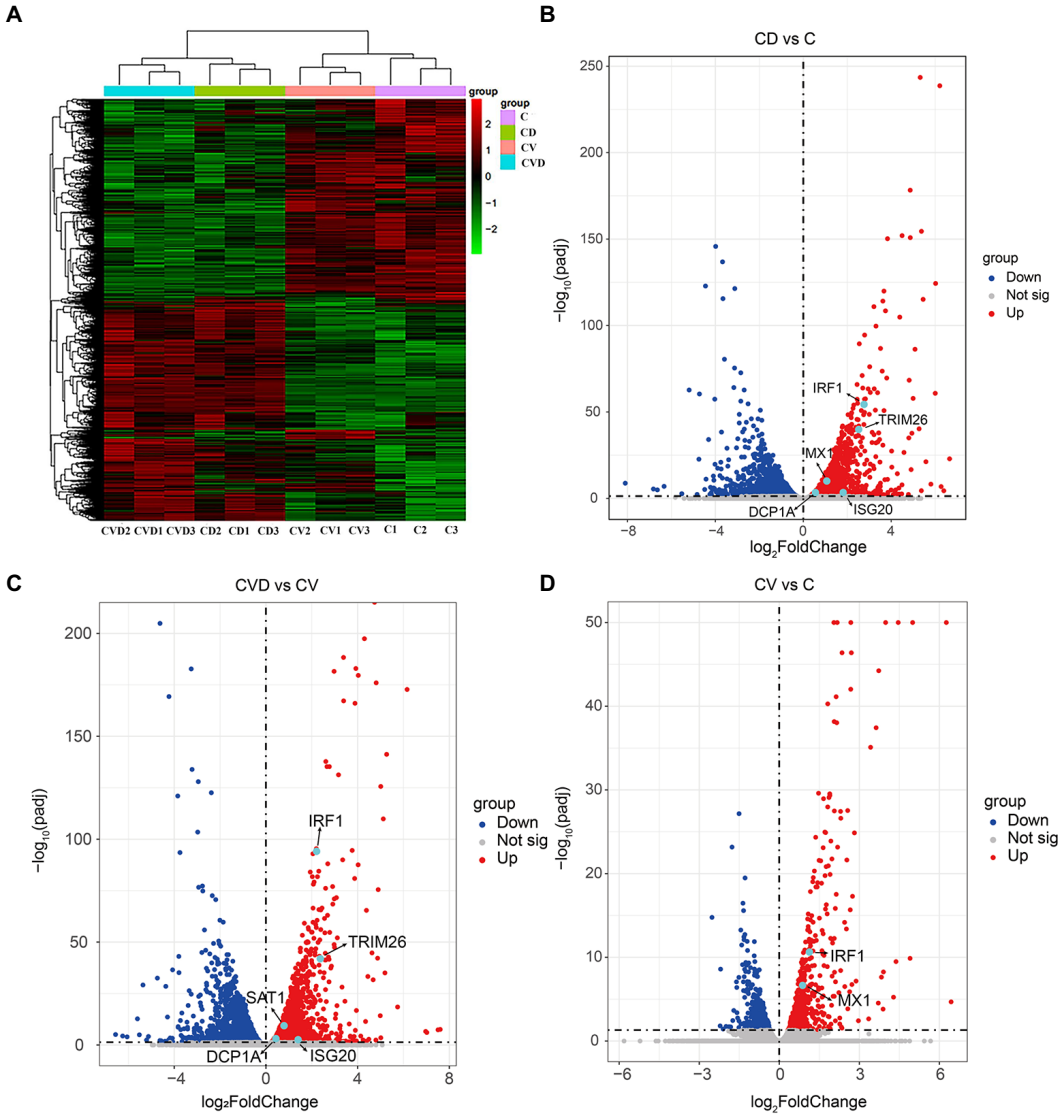
Heat map and volcano map of differentially expressed genes obtained by high-throughput sequencing of samples from different treatment groups. **(A)** Heap map of differentially expressed genes in different treatment groups. **(B)** Volcano map of differentially expressed genes in the CD group (PAMs incubated with TSN) compared with the C group (mock). **(C)** Volcano map of differentially expressed genes in the CVD group (ASFV-infected PAMs incubated with TSN) compared with the CV group (ASFV-infected PAMs). **(D)** Volcano map of differentially expressed genes in the CV group compared with the C group.

To explore the effect of TSN on host genes in PAMs, high-throughput sequencing was performed. Clustering analysis of differentially expressed genes in the four subgroups, i.e., the mock group (C group), the group of PAMs incubated with TSN (CD group), the group of ASFV-infected PAMs (CV group) and the group of ASFV-infected PAMs incubated with TSN (CVD group), showed that the three replicate samples of each treatment group were successfully clustered together, which fully illustrated the accuracy of sampling (Figure 8A). Comparative analysis was performed between different treatment groups, and a total of 3 sets of comparative analysis were performed, i.e., CD group vs. C group; CVD group vs. CV group; and CV group vs. C group. The normalized RPKM values obtained by applying the edgeG package were subjected to differential genetic analysis between samples, and volcano plots were plotted for each comparison group according to Fold-change (fold of expression difference) as well as *p* values (*p* < 0.05, Fold-change ≥2, FDR < 0.05). As shown in Figure 8B, a total of 2,196 up-regulated genes were screened in the CD group compared with the C group, such as the IRF1, TRIM26, MX1 and so on; as shown in Figure 8C, a total of 2,504 up-regulated genes were screened in the CVD group compared with the CV group, such as the IRF1, TRIM26 and so on; as shown in Figure 8D, a total of 600 upregulated genes were screened in the CV group compared with the C group, such as the IRF1, MX1 and so on.

### Effects of TSN on the mRNA expression level of IFNs and 9 ISGs in PAMs by RT-qPCR

**Figure 9:**
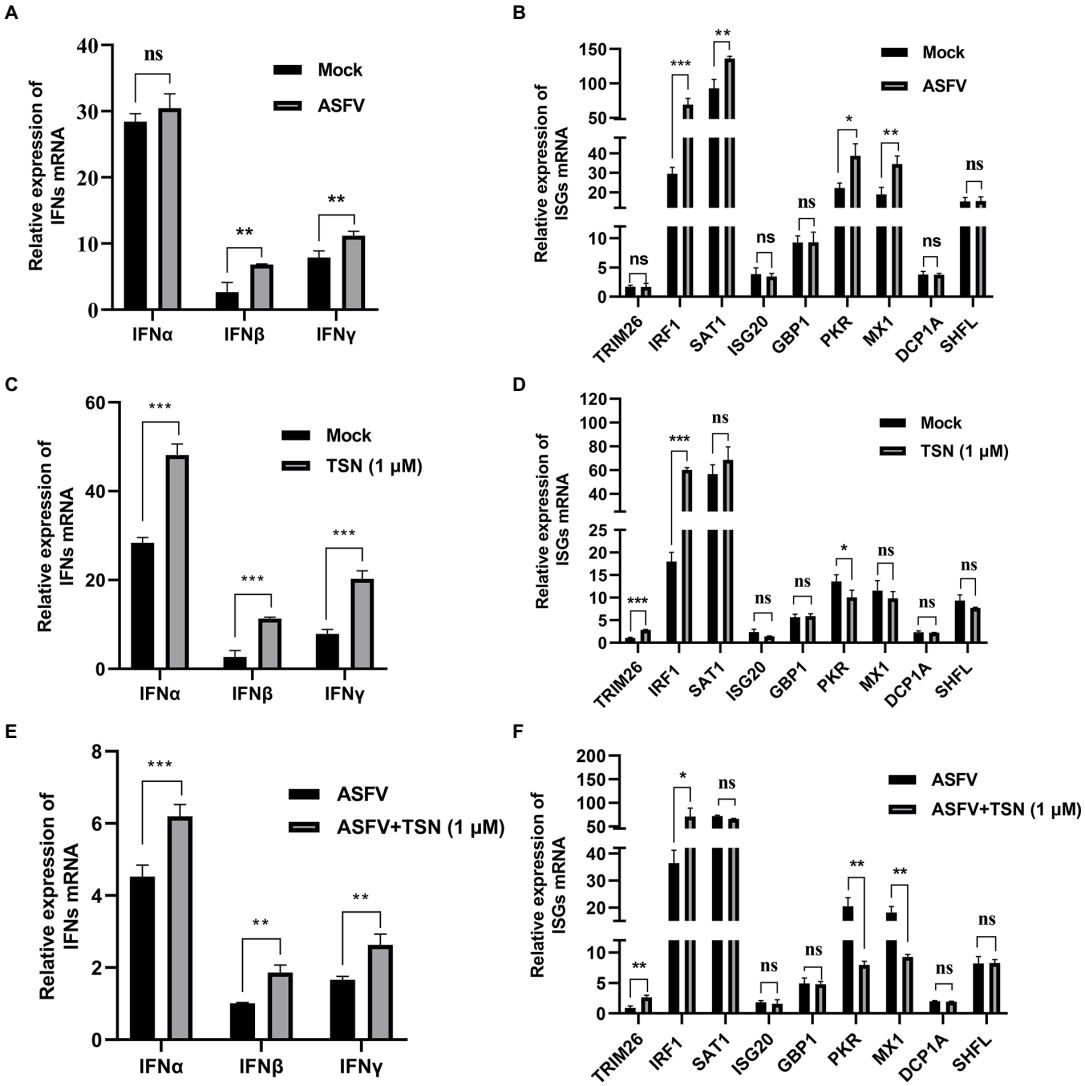
Effects of TSN on the mRNA expression of IFNs and 9 ISGs in PAMs. 0.014 MOI ASFV infected the PAMs for 24 h to test its effect on the expression level of IFNs (IFNα, IFNβ and IFNγ) and 9 ISGs (TRIM26, IRF1, SAT1, ISG20, GBP1, PKR, MX1, DCP1A and SHFL). Besides, 1 μM TSN was added to PAMs with or without ASFV infection (0.014 MOI) for 24 h to test its effect on the expression of IFNs (IFNα, IFNβ and IFNγ) and 9 ISGs (TRIM26, IRF1, SAT1, ISG20, GBP1, PKR, MX1, DCP1A and SHFL). The vertical axis shows the relative expression level (fold-change) of IFNs or 9 ISGs mRNA. The fold-changes were calculated by the 2^∆∆CT^ method and normalized by IFNβ level **(A,C,E)** or TRIM26 level **(B,D,F)** in mock or ASFV group. **(A)** The mRNA level of IFNα, IFNβ and IFNγ in PAMs with ASFV infection was analyzed using RT-qPCR. **(B)** The mRNA level of 9 ISGs in PAMs with ASFV infection was analyzed using RT-qPCR. **(C)** The mRNA levels of IFNα, IFNβ and IFNγ in PAMs with or without TSN treatment were analyzed using RT-qPCR. **(D)** The mRNA levels of 9 ISGs in PAMs with or without TSN treatment were analyzed using RT-qPCR. **(E)** The mRNA levels of IFNα, IFNβ and IFNγ in ASFV-infected PAMs with or without TSN treatment were analyzed using RT-qPCR. **(F)** The mRNA levels of 9 ISGs in ASFV-infected PAMs with or without TSN treatment were analyzed using RT-qPCR. ^***^ denotes *p* < 0.001; ^**^ denotes *p* < 0.01; ^*^ denotes *p* < 0.05; ns denotes not significant.

Firstly, we explored the effect of ASFV infection on the expression levels of IFNs (IFNα, IFNβ and IFNγ) and 9 ISGs (TRIM26, IRF1, SAT1, ISG20, GBP1, PKR, MX1, DCP1A, and SHFL) in PAMs infected with ASFV for 24 h by RT-qPCR. Results showed that ASFV infection could significantly upregulate the mRNA level of IFNβ and IFNγ (Figure 9A), and also could significantly upregulate the mRNA level of IRF1, SAT1, PKR and MX1 (Figure 9B). To explore whether TSN exerts its anti-ASFV activity *via* inducing the expression of IFNs and ISGs, the PAMs were treated with 1 μl TSN for 24 h and then measured the expression levels of IFNs (IFNα, IFNβ, and IFNγ) and 9 ISGs (TRIM26, IRF1, SAT1, ISG20, GBP1, PKR, MX1, DCP1A, and SHFL) by RT-qPCR. As shown in Figure 9C, TSN treatment could significantly increase the mRNA levels of IFNα, IFNβ, and IFNγ. Whereas, TSN treatment resulted in differentiated effects on 9 ISGs expression, it increased the mRNA expression levels of IRF1, TRIM26, and SAT1, while significantly decreased the mRNA expression levels of PKR in PAMs (Figure 9D). Next, we examined the effects of TSN on the expression of related IFNs and ISGs in PAMs infected with ASFV. As shown in Figure 9E, TSN treatment significantly increased the mRNA levels of IFNα, IFNβ, and IFNγ in PAMs infected with ASFV. In ASFV infected PAMs, TSN treatment also resulted in differentiated effects on 9 ISGs expression, it significantly increased the mRNA level of IRF1 and TRIM26, while decreased the mRNA levels of PKR and MX1 (Figure 9F). These results showed that TSN could upregulate the mRNA levels of 2 ISGs including IRF1, TRIM26. Therefore, using transcriptome sequencing and RT-qPCR methods, we found that TSN stably upregulates both IRF1 and TRIM2 in PAMs.

### TSN upregulates the mRNA expression levels of IFNs and IRF1 and the protein level of IRF1 in PAMs

**Figure 10:**
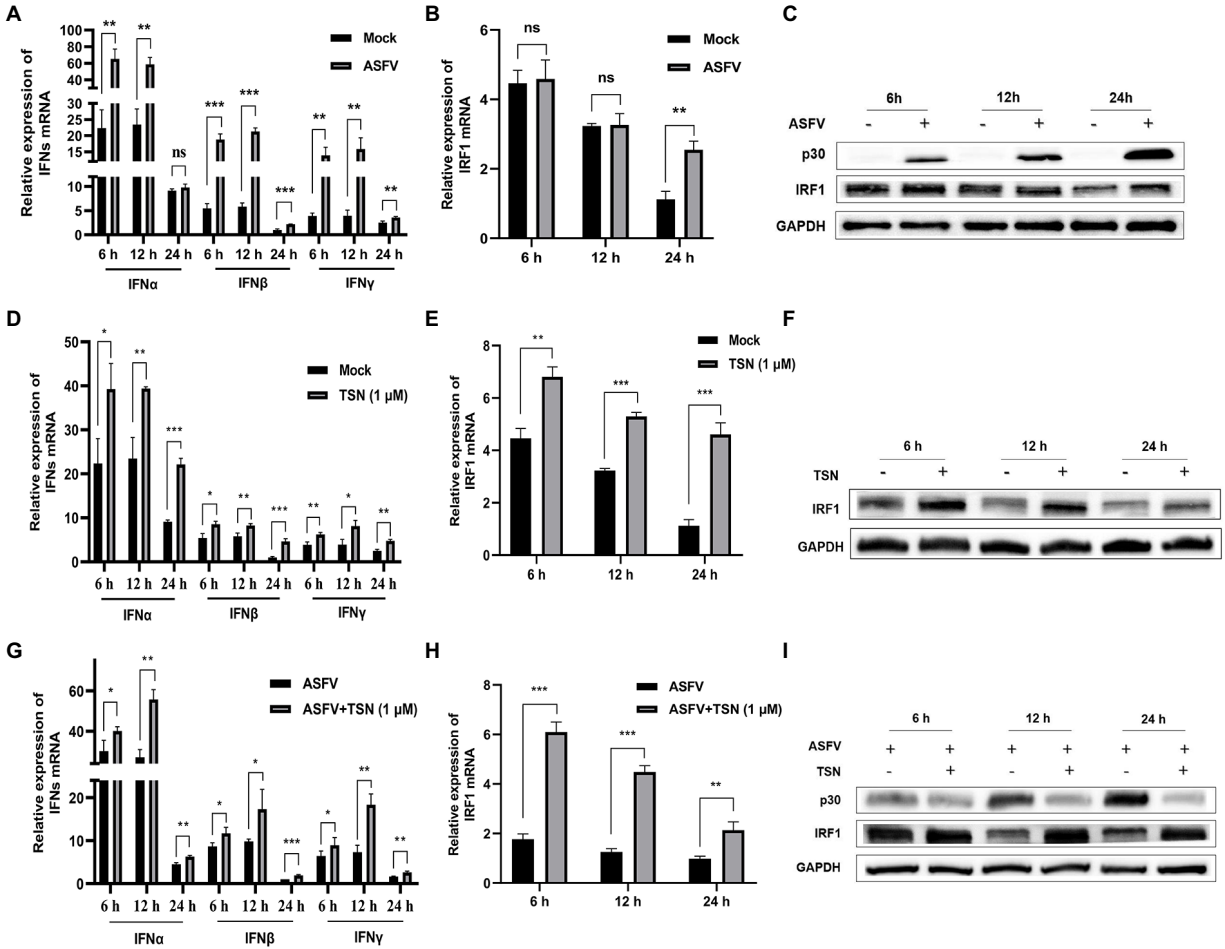
TSN treatment upregulates the mRNA expressions of IFNs and IRF1, and the protein expression of IRF1 in PAMs. 0.014 MOI ASFV infected the PAMs for 6, 12, and 24 h to test its effect on the expression level of IFNs (IFNα, IFNβ and IFNγ) and IRF1. Besides, 1 μM TSN was added to PAMs with or without ASFV infection (0.014 MOI) for 6, 12, and 24 h to test its effect on the expression levels of IFNs (IFNα, IFNβ and IFNγ) and IRF1. The vertical axis shows the relative expression level (fold-change) of IFNs or IRF1 mRNA. The fold-changes were calculated by the 2^∆∆CT^ method and normalized by IFNβ or IRF1 levels at 24 h in mock or ASFV group. **(A)** The mRNA levels of IFNα, IFNβ and IFNγ in PAMs with ASFV infection at 6, 12, and 24 h was analyzed using RT-qPCR. **(B)** The mRNA level of IRF1 in PAMs with ASFV infection at 6, 12, and 24 h was analyzed using RT-qPCR. **(C)** The protein level of IRF1 in PAMs with ASFV infection at 6, 12, and 24 h was analyzed using Western Blot. **(D)** The mRNA levels of IFNα, IFNβ and IFNγ in PAMs with or without TSN treatment at 6, 12, and 24 h was analyzed using RT-qPCR. **(E)** The mRNA level of IRF1 in PAMs with or without TSN treatment at 6, 12, and 24 h was analyzed using RT-qPCR. **(F)** The protein level of IRF1 in PAMs with or without TSN treatment at 6, 12, and 24 h was analyzed using Western Blot. **(G)** The mRNA levels of IFNα, IFNβ and IFNγ in ASFV-infected PAMs with or without TSN treatment at 6, 12, and 24 h was analyzed using RT-qPCR. **(H)** The mRNA level of IRF1 in ASFV-infected PAMs with or without TSN treatment at 6, 12, and 24 h was analyzed using RT-qPCR. **(I)** The protein level of IRF1 in ASFV-infected PAMs with or without TSN treatment at 6, 12, and 24 h was analyzed using Western Blot. ^***^ denotes *p* < 0.001; ^**^ denotes *p* < 0.01; ^*^ denotes *p* < 0.05; ns denotes not significant.

As shown in Figure 10A, ASFV infection significantly upregulated the mRNA level of IFNα at 6 h and could significantly upregulate the mRNA level of IFNβ and IFNγ at 6, 12, and 24 h. Figures 10B,C showed that the mRNA and protein levels of IRF1 were significantly upregulated in PAMs infected with ASFV at 24 h, respectively. Then, we further investigated the effects of TSN on the mRNA expression levels of IFNα, IFNβ, IFNγ, IRF1 and the protein level of IRF1 in PAMs with or without ASFV infection at 6, 12, and 24 h post TSN treatment. As shown in Figure 10D, TSN treatment in PAMs significantly upregulated the mRNA levels of IFNα, IFNβ, and IFNγ at 6, 12, and 24 h. Besides, TSN treatment in PAMs significantly upregulated the mRNA level (Figure 10E) and the protein level (Figure 10F) of IRF1 at 6, 12, and 24 h. Similarly, the TSN treatment significantly increased the mRNA levels of IFNα, IFNβ, and IFNγ (Figure 10G), as well as the mRNA level (Figure 10H) and the protein level (Figure 10I) of IRF1 in ASFV-infected PAMs at 6, 12, and 24 h. These results suggest that IRF1 might play a key role in anti-ASFV activity of TSN.

### TSN suppresses ASFV replication in PAMs by upregulating IRF1 expression

**Figure 11:**
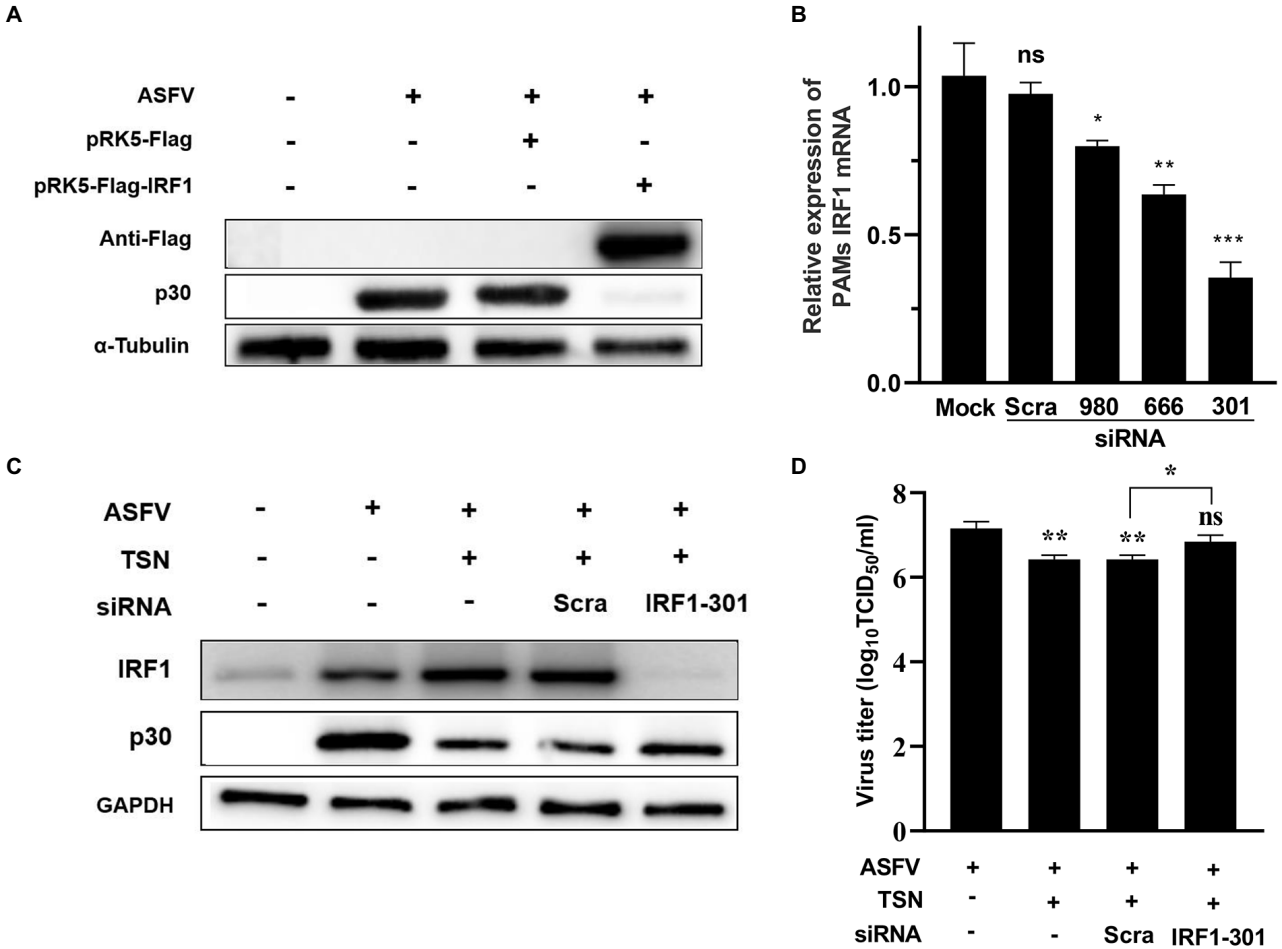
TSN suppresses ASFV replication *via* upregulating IRF1 expression. **(A)** PK-15 cells were challenged with 0.014 MOI ASFV at 37°C for 2 h, then PRK5-FLAG-IRF1 was transfected into PK15 cells at 37°C for 6 h. After that, the supernatant was discarded and the cells were washed with PBS. Then fresh maintenance medium was added in PK-15 cells and incubated at 37°C for 16 h. The samples were harvested at 16 h post transfection for Western Blot. **(B)** PAMs were transfected with 2 μg/ml of three different siRNA against IRF1 and scramble siRNA (siRNA-Scra) at 37°C for 4 h. After that, the supernatant was discarded and the cells were washed with PBS, then fresh maintenance medium was added in PAMs. IRF1 was detected by RT-qPCR at 24 h post siRNA transfection. **(C)** PAMs were challenged with 0.014 MOI ASFV at 37°C for 2 h, then the siRNA-IRF1-301 or siRNA-Scra was transfected into PAMs at 37°C for 6 h. After that, the supernatant was discarded and the cells were washed with PBS followed by treatment with 1 μM TSN. After culture at 37°C for 16 h, the samples were collected for Western Blot. **(D)** The samples from panel **(C)** were collected for endpoint dilution method analysis. ^***^ denotes *p* < 0.001; ^**^ denotes *p* < 0.01; ^*^ denotes *p* < 0.05. ns denotes no significant difference.

Further experiments were conducted to confirm that TSN plays an antiviral role by upregulating the expression of IRF1. As shown in Figure 11A, When PK-15 infected with ASFV were transfected with the overexpression plasmid of IRF1 (pPK15-Flag-IRF1), ASFV replication was significantly inhibited, reflected by decreased expression of viral p30 protein compared with that in ASFV-infected PK-15 cells. In addition, three different siRNAs (siRNA-IRF1-301, -666 and -980) were designed and used to silence the expression of the endogenous IRF1 of PAMs. The results from RT-qPCR showed that of siRNAs tested siRNA-IRF1-301 exhibited the most potent silencing efficacy on IFR1 expression (Figure 11B). Consistently, TSN treatment significantly upregulated the protein expression of IRF1 in ASFV-infected PAMs while inhibited ASFV replication, reflected by a significantly decrease in ASFV p30 expression. However, when IRF1 was silenced by siRNA-IRF1-301, the protein expression of IRF1 was significantly decreased while viral p30 expression was significantly increased (Figure 11C). In addition, the virus titer of ASFV was significantly increased when IRF1 was silenced by siRNA-IRF1-301 (Figure 11D), which indicate that the inhibition of TSN on ASFV infection is reversed by IRF1 silencing. These results demonstrate that TSN suppresses ASFV infection *via* upregulating the expression of cellular IRF1.

## Discussion

ASF is a viral disease characterized by high mortality that affects domestic swine and wild pigs (Oganesyan et al., 2013). Currently, there is no safe and effective vaccine against ASFV available in the world. Therefore, the development of effective drugs against ASFV is especially important to prevent and control the disease. Although several compounds were reported to have *in vitro* anti-ASFV activity, the *in vivo* antiviral efficacy of these compounds has not been validated other than interferons (Keita et al., 2010; Arabyan et al., 2019). Therefore, it is very urgent to identify new effective antiviral drugs for controlling ASFV (Huang et al., 2021). TSN was used in the clinics as anthelmintic to expel parasites from alimentary tract in China (Shi and Li, 2007). It possesses numerous biological activities (He et al., 2010; Jin et al., 2017), while its anti-ASFV activity has not been reported. Here, we reported for the first time that TSN exhibited strong anti-ASFV efficacy at sub-micromolar concentrations in PAMs. Furthermore, we reveal that TSN inhibited ASFV infection by upregulating cellular IRF1 rather than direct inactivating virions.

Alveolar macrophages (AM) are lung-resident macrophages representing more than 90% of immune cells in bronchoalveolar lavage in mammals and provide the main immune response against respiratory infectious (Tatoyan et al., 2020). PAMs are the primary cells for the efficient propagation of ASFV and are a good model for studying anti-ASFV drugs. In this study, PAMs were chosen to explore the antiviral effect of TSN against ASFV. Using a well-established *in vitro* infection model of ASFV p72 genotype II GZ201801-38 strain, we found that TSN exhibited a potent inhibitory effect against ASFV replication in PAMs in a dose-dependent manner. It possesses a stable antiviral effect that does not vary with changes in the dose of ASFV infection. Importantly, the antiviral EC_50_ of TSN is 0.085 μM, which is much lower than the concentrations of other effective anti-ASFV compounds that have been reported. For example, genkwanin, genistein and GS-441524 were reported to inhibit ASFV infection with EC_50_ of 2.9, 13, and 287.5 μM, respectively (Arabyan et al., 2018; Hakobyan et al., 2019; Huang et al., 2021), which are much less anti-ASFV efficacy than TSN. The very low EC_50_ means that antiviral efficacy can be obtained at very low dosing concentrations, thus making it more likely that the compound will exhibit *in vivo* antiviral activity under conditions of no toxicity to the animals.

To define the mechanism of action by which TSN inhibits ASFV infection, we conducted a series of experiments including different TSN treatment modes, ASFV binding assay, internalization assay, release assay and direct interaction assay. The results showed that TSN does not affect viral attachment and release, while it affects viral internalization. In addition, TSN treatment does not directly interact with ASFV. These results together suggest that TNS plays anti-ASFV role not by reducing the sensitivity of PAMs to ASFV infection or by direct inactivating virions. Interestingly, TSN showed a remarkable inhibition on ASFV infection under the pre-treatment mode in a time and dose-dependent manner, suggesting that TSN inhibits ASFV likely by regulating host genes in PAMs.

Previous studies demonstrated that IFNs have antiviral effects, especially type I IFN (IFNα and IFNβ) has broad-spectrum antiviral effects. At the same time. IFNα can strongly modulate the innate and adaptive immune response in the host (Vancott et al., 2003; Loo et al., 2006; Frias et al., 2012; Gibbert et al., 2013). It was also reported that the secretion of IFNγ could control viral replication (Whitman et al., 2009). In 2020, it was reported that porcine type I and type II IFNs could inhibit ASFV replication both *in vivo* and *in vitro* conditions (Fan et al., 2020). Watanabe et al. reported that TSN and IFNα co-treatment can show synergistic antiviral effect on HCV by inducing the activation of a component of interferon stimulating gene factor 3 (ISGF3), which enhances the inhibitory effect of IFNα (Watanabe et al., 2011). The results in our study verified that TSN treatment could significantly upregulate the mRNA level of type I IFN (IFNα and IFNβ) and type II IFN (IFNγ) in PAMs, illustrating that the antiviral effect of TSN in PAMs was likely related to the IFNs. TSN-induced IFNs production may have positive implications against ASFV, including less likely to induce resistance and effective against different subtypes of ASFV strains.

To further find out which ISG regulated by IFNs plays the ultimate antiviral role of TSN, we detected the ISGs mRNA level in PAMs by RNA sequencing and RT-qPCR methods. Our results showed that no matter whether PAMs were infected with ASFV or not, once treated with TSN, the mRNA level of ISG IRF1 was significantly and stably upregulated in PAMs. At the same time, the protein level of IRF1 was also significantly upregulated in PAMs by TSN treatment. IRF1 was the first member of the IRF family to be recognized and was originally discovered as a transcriptional activator of IFNβ (Fujita et al., 1988). It was reported that IRF1 has physiological roles in tumor prevention, cytokine signaling, and cell growth regulation (Kroger et al., 2002). In recent years, IRF1 was shown to participate in the host defense system and play a role in resisting viral invasion. IRF1 has been reported to significantly inhibit several clinically important viruses, such as vesicular stomatitis virus, influenza virus, gamma herpes virus, hepatitis C virus, yellow fever virus, West Nile virus and human immunodeficiency virus type 1 (Schoggins et al., 2014; Mboko et al., 2017; Panda et al., 2019; Feng et al., 2021). It was interesting to figure out whether the efficacy of TSN against ASFV is related to the upregulation of IRF1. Our results showed that overexpression of IRF1 possesses anti-ASFV activity and results from siRNA assay demonstrate that TSN suppresses ASFV infection *via* upregulating the expression of cellular IRF1. The results of the present study suggest that the endogenous IFNs induction through upregulating IRF1 might be an effective pathway for inhibiting the replication of ASFV, and TSN might be a promising anti-ASFV agent as an IFNs inducer.

In this study, we demonstrated that TSN inhibited ASFV internalization, meanwhile, TSN suppressed ASFV replication *via* activating the expression of cellular IRF1. Given that both the events of ASFV internalization and IRF1 activation occurs at early stage of ASFV infection, it is possible that IRF1 inhibits ASFV internalization. However, the relationship between IRF1 and ASFV internalization need to be disclosed based on further investigations. Jin and colleagues showed that TSN inhibits influenza A virus infection at an early stage by altering PA protein nuclear localization (Jin et al., 2019). Our results suggest that TSN played its antiviral role by upregulating the host gene IRF1. However, genes of ASFV code at least 150 proteins, and the pathogenesis of ASFV is quite complicated. Whether other mechanisms of action allow TSN to exert antiviral effect against ASFV infection still deserves further exploration.

In summary, our study shows that TSN potently suppresses ASFV replication at sub-micromolar concentrations. Furthermore, we have elucidated the mechanisms underlying antiviral effect of TSN against ASFV infections as upregulating the ISG IRF1 of the host, which suggests that IRF1 might be a valuable target for combating ASFV. It may be particularly advantageous to use TSN as an anti-ASFV agent, especially considering that it was used as an ascarifuge with acceptable biological safety. Further *in vivo* studies will be necessary to confirm TSN as a novel and effective ASFV inhibitor in swine.

## Data availability statement

The data presented in the study are deposited in the GEO respository, accession number GSE206118.

## Author contributions

JC, WQ, and CW designed the study. YL and XZ wrote the manuscript. YL, XZ, and ZL performed the experiments and analyzed the results. LH, XL, GZ, and WJ supervised the project and critically revised the manuscript. All authors contributed to the article and approved the submitted version.

## Funding

This work was funded by the National Natural Science Foundation of China (grant numbers 31941019 and 31872521), and the Basic Research & Applying Basic Research Foundation of Guangdong Province (grant number 2019B1515210007).

## Conflict of interest

The authors declare that the research was conducted in the absence of any commercial or financial relationships that could be construed as a potential conflict of interest.

## Publisher’s note

All claims expressed in this article are solely those of the authors and do not necessarily represent those of their affiliated organizations, or those of the publisher, the editors and the reviewers. Any product that may be evaluated in this article, or claim that may be made by its manufacturer, is not guaranteed or endorsed by the publisher.
